# Efavirenz Is Not Associated with an Increased Risk of Depressive Disorders in Patients Living with HIV: An 11-Year Population-Based Study in Taiwan

**DOI:** 10.3390/healthcare9121625

**Published:** 2021-11-24

**Authors:** Chia-Wen Li, Yen-Chin Chen, Nan-Yao Lee, Po-Lin Chen, Ming-Chi Li, Chung-Yi Li, Wen-Chien Ko, Nai-Ying Ko

**Affiliations:** 1 Division of Infectious Diseases, Department of Internal Medicine, National Cheng Kung University Hospital, College of Medicine, National Cheng Kung University, Tainan 704, Taiwan; li.cw29@gmail.com (C.-W.L.); nanyao@mail.ncku.edu.tw (N.-Y.L.); plin@ncku.edu.tw (P.-L.C.); asurangle@gmail.com (M.-C.L.); winston3415@gmail.com (W.-C.K.); 2 Center for Infection Control, National Cheng Kung University Hospital, College of Medicine, National Cheng Kung University, Tainan 704, Taiwan; 3 Department of Public Health, College of Medicine, National Cheng Kung University, Tainan 701, Taiwan; 4 Department of Medicine, College of Medicine, National Cheng Kung University, Tainan 701, Taiwan; 5 Department of Nursing, National Cheng Kung University Hospital, College of Medicine, National Cheng Kung University, Tainan 704, Taiwan; yenchin2427@gmail.com; 6 Department of Public Health, College of Public Health, China Medical University, Taichung 406, Taiwan

**Keywords:** cohort studies, human immunodeficiency virus, active antiretroviral therapy, depressive disorder, Cox proportional hazard model

## Abstract

(1) Background: This study aimed to determine the association between the use of efavirenz and depressive disorders among human immunodeficiency virus (HIV)-infected patients. (2) Methods: A retrospective cohort study was conducted using Taiwan’s National Health Insurance Database. We identified patients receiving anti-retroviral therapy (ART) between 2000 and 2009; these patients were followed until 2010 for diagnoses of depressive disorders using the Cox proportional hazard model to estimate hazard ratios. (3) Results: After up to 11 years of follow-up, the incidence of depressive disorders for the efavirenz-treated group was estimated at 12.2/1000 person-years (PYs), and the control group was at 12.5/1000 PY (*p* = 0.822). The independent risk factors for depressive disorders included an insurance premium of less than NTD 17,820 (New Taiwan Dollars—NTD) (adjusted hazard ratio (aHR) 2.59, 95% confidence interval (CI), 1.79–3.76, *p* < 0.001), and between NTD 17,821 and NTD 26,400 (aHR 1.55, 95% CI, 1.04–2.31, *p* = 0.030), living in Southern Taiwan (aHR 1.49, 95% CI, 1.21–1.84, *p* = 0.002), and with a psychiatric history (excluding depressive disorders) (aHR 4.59, 95% CI, 3.51–6.01, *p* = 0.030). (4) Conclusions: This study concluded that ART-treated patients with a past history of psychiatric disorders, lower insurance premium, and living in Southern Taiwan have an increased risk of depressive disorders, which are not associated with the use of efavirenz.

## 1. Introduction

Human immunodeficiency virus (HIV)-infected individuals have been found to experience a higher prevalence of psychiatric disorders, with rates exceeding those of the general population [1,2]. A nationwide population-based study conducted in Taiwan noted that the three leading psychiatric disorders in HIV-infected individuals included anxiety disorders, depressive disorders, and alcohol/substance use [3]. Depression is one of the major challenges facing people living with HIV [4]. Previous studies have shown varying prevalence of depression in HIV-infected individuals in different parts of the world, ranging from 12% to 60% [5,6,7,8,9]. Socioeconomic disadvantage has been noted to increase the risk of depression among subgroups of the HIV-infected population in a Brazilian study [10]. The incidence of depressive disorders has also increased with age and over calendar time among people living with HIV [11]. Meanwhile, depression also reduces the adherence to treatment and impairs the effectiveness of anti-retroviral therapy (ART), leading to disease progression and mortality [12,13].

With the introduction of the highly active anti-retroviral therapy (HAART), HIV infection has been transformed into a chronic condition for which lifelong medication is necessary for disease control and better chances of survival, but chronic depression/anxiety could still be observed among nearly one-fourth of HIV-infected patients [14]. Efavirenz has been widely used, and the most commonly reported side effects were a reduction in sleep quality, depression, dizziness, and anxiety [15]. However, its association with brain physiology or depression is still unclear [16]. An animal study of rats showed an acute anxiogenic-like effect from treatment with efavirenz, and sub-chronic effects on both anxiogenic-like and depressive-like behaviors [17]. A study showed that efavirenz was not a risk factor for depressive symptoms among HIV-infected patients without a history of depressive disorders; rather, it was associated with a risk of depressive symptoms five times higher in one year among patients with a history of depressive disorders, and the difference in risk was especially obvious after 24 weeks of follow-up [18]. A meta-analysis of data from several clinical trials including 5332 patients revealed that the hazard ratio (HR) of the events of suicidal ideation and attempt was 2.1 among patients taking an efavirenz-containing regimen; the highest HR (3.69) was observed in the first 24 weeks [19]. This particular study indicated that patients taking efavirenz tended to be susceptible to an increased risk of developing depressive symptoms, including suicidal ideation and attempt.

However, the long-term impacts of efavirenz on depressive disorders have not been well-elucidated. A Spanish cohort argued that the use of ART, regardless of the regimens, would significantly decrease the incidence of such depressive disorders and did not conclude the positive association of efavirenz with an increased risk of clinically significant depression, including depression requiring drug therapy and suicide attempt [20]. A Danish study showed that the utilization of anti-depressants was not significantly different between patients who were and were not taking efavirenz over a 14-year follow-up [11]. Hence, the long-term impacts of efavirenz on depressive disorders have been neither comprehensive nor consistent.

Therefore, this retrospective study was aimed at further evaluating the association between the use of efavirenz and the occurrence of depressive disorders among HIV-treated patients.

## 2. Materials and Methods

### 2.1. Data Source

The data analyzed were retrieved from Taiwan’s National Health Insurance Database (NHIRD), which covered medical claims of approximately 99% of the 23 million people of Taiwan and included 99% of the hospitals and clinics under a single-payer health insurance system [21]. The NHIRD included the original medical and pharmacy records of all patients. A recent study has further validated the utility of the database for identifying HIV-related diseases [3]. The study was approved by the Institutional Review Board of National Cheng Kung University Hospital (No. B-BR-103-056).

### 2.2. Study Population and Design

**Figure 1:**
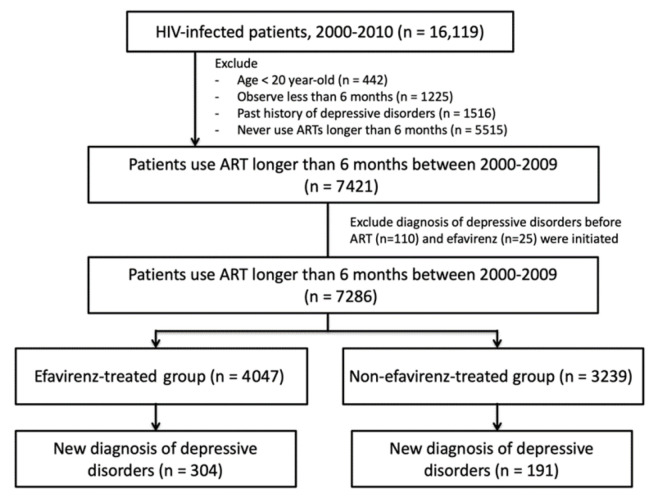
Flowchart of study patients’ enrollment and follow-up.

Data for the HIV-infected patients were identified by the International Classification of Diseases version 9 (ICD-9) codes (042, V08) from the claim data in the period of 2000–2010 (Figure 1). Information on a total of 16,119 HIV-infected patients was extracted from NHIRD from 2000 to 2010. Patients who were under 20 years old were excluded (*n* = 442). Patients who stayed in the insurance registry for less than 6 months (*n* = 1225), who had a diagnosis of depressive disorders (ICD-9 296.2, 296.3, 311) before HIV diagnosis (*n* = 1516), who had not been on ART for more than 6 months (*n* = 5515), were also excluded from the analysis. We further excluded those with diagnoses of depressive disorders before the initiation of ART (*n* = 110) and efavirenz (*n* = 25). The remaining 7286 patients who had already been prescribed efavirenz were classified as the efavirenz-treated group (*n* = 4047), while patients who had never been prescribed efavirenz during the study period were classified as the non-efavirenz-treated group (*n* = 3239).

This was a retrospective cohort study in which the date of starting follow-up (index date) for the efavirenz-treated and control group was respectively the date of the initial prescription of efavirenz and the date of the first prescription of ART for treating HIV infection in 2000–2009. Exclusion of the time period between the date of HIV diagnosis and the date of efavirenz prescription was made to minimize the potential for immortal time bias [22]. All the study subjects were followed from the index date to the occurrence of the endpoint or censoring.

### 2.3. Research Variables

The endpoint was recognized to have been reached when the diagnosis of depressive disorders (ICD-9 codes: 296.2, 296.3, or 311) appeared in the outpatient or inpatient claims during the follow-up period. Demographic characteristics and clinical risk factors for depression were also retrieved from the claims data, including the age at HIV diagnosis, gender, geographic area of residence, monthly income for determining insurance premium, and selected comorbidities. The geographic area of residence was classified into North, Central, South, East, and remote islands. The comorbidities included opportunistic infections (based on ICD-9: 003, 007.4, 008.8, 018,031.2, 053.9, 054, 078.5, 112, 117.9, 130, 136.3, 136.9, 321, 482.9, 799.4), hypertension (ICD-9: 405), diabetes mellitus (ICD-9: 250), cardiovascular diseases (ICD-9: 272, 273.2, 414.9, 429.2, 429.9, 440.9), and past history of psychiatric diseases other than depressive disorders (ICD 9: 295, 296.1, 296.9, 297, 300, 305). The above selected comorbidities were determined when the corresponding diagnoses were listed in 3 or more inpatient or outpatient claims in our dataset, regardless of which specialists had made such diagnoses.

### 2.4. Statistical Analysis

SAS 9.3 for Windows (SAS institute Inc., Cary, NC, USA) was used to analyze the data. To compare the characteristics between efavirenz-treated patients and controls, the Chi-square test was utilized to compare categorical variables, and the Analysis of Variance (ANOVA) was utilized to compare continuous variables. The incidence rates of depressive symptoms, which were based on the Poisson assumption, were calculated as the number of patients who encountered the study end-point over the total person-years accumulated throughout the study period, and were expressed per 1000 person-years. Cox proportional hazard models were built to assess the associations of depressive symptoms in relation to efavirenz and other potential predictors for depressive symptoms. All hypothesis testings were two-tailed and considered a *p* value of less than 0.05 as statistically significant.

### 2.5. Sensitivity Analysis

To address the potential information bias, we further set 3 scenarios for sensitivity analyses. First, we excluded those patients who developed depressive disorders more than one month after ARTs were discontinued. Additionally, the patients whose efavirenz was replaced by other ARTs during the follow-up period were also excluded in this scenario. Second, the efavirenz-treated patients who discontinued prescriptions for more than 6 months were excluded. In the third scenario, only those patients whose depressive disorders were diagnosed by psychiatrists were counted in the analysis.

## 3. Results

Out of 7286 patients, 4047 (55.5%) individuals who had been given efavirenz at least once as part of ART were categorized as the efavirenz-treated group, while the other 3239 (44.5%) patients who had never been given efavirenz were then categorized as the non-efavirenz-treated group. The detailed demographic characteristics are listed in Table 1. In both groups, around 93% of patients were male, with an approximate mean age of 35 years old at HIV diagnosis. In the efavirenz-treated group, 2109 (52.1%) patients were from Northern Taiwan, while 2037 (62.9%) patients in the non-efavirenz-treated group were from Northern Taiwan. Nearly half of patients from both groups had an insurance premium based on a monthly income level of less than NTD 17,280. In total, 2268 (56%) patients in the efavirenz-treated group were diagnosed as having opportunistic infections, while only 1675 (51.7%) patients in the non-efavirenz-treated group had the same diagnosis (*p* < 0.001). As for past psychiatric disorders other than depressive disorders, 239 (5.9%) in the efavirenz-treated group and 208 (6.4%) patients in the non-efavirenz-treated group had such disorders (*p* = 0.362). The average observational time was 6.1 years (range: 0–11 years, standard deviation (SD): 3.9) for the efavirenz-treated group and 4.7 years (range 0–11 years, SD: 3.16) for the non-efavirenz-treated group (*p* < 0.001).

Among the 495 patients who were newly diagnosed as having depressive disorders after initiation of efavirenz or other ART, 304 patients (7.5%) were from the efavirenz-treated group, while the other 191 patients (5.9%) were from the non-efavirenz-treated group (*p* < 0.001). The incidence rate of depressive disorders among the efavirenz-treated group was estimated at 12.2/1000 person-year (PY), which was not significantly lower than that of the non-efavirenz-treated group (12.5/1000 PY) (*p* = 0.822). The majority of patients (*n* = 376, 76%) were diagnosed as having depressive disorders by psychiatrists, and 50 (10%) patients received a diagnosis for depressive disorders by physicians of internal medicine.

Table 2 shows the Cox proportional hazard model that estimated the crude and adjusted hazard ratio (aHR) of depressive disorders. The use of efavirenz was not significantly associated with the occurrence of depressive disorders (aHR 0.92, 95% CI, 0.76–1.10, *p* = 0.353) after adjustment for potential confounders. The independent risk factors significantly associated with the occurrence of depressive disorders included: patients with a past history of psychiatric disorders (aHR 4.59, 95% CI, 3.51–6.0, *p* < 0.001), patients’ monthly income level of less than NTD 17,280 as opposed to those with a monthly income of more than NTD 43,901 (aHR 2.59, 95% CI, 1.79–3.76, *p* < 0.001). The aHR was also significantly increased for those with a monthly income of NTD 17281–26400 (1.55, 95% CI, 1.04–2.31, *p* = 0.030). Living in Southern Taiwan was also a significant risk factor for the onset of depressive disorders, with an aHR of 1.49 (95% CI, 1.21–1.84, *p* = 0.002), as compared to those living in Northern Taiwan. On the other hand, having opportunistic infections prior to ART initiation was significantly associated with a reduced aHR at 0.80 (95% CI, 0.67–0.95, *p* = 0.011).

Results from the sensitivity analyses according to three scenarios are listed in Table 3. In the first two scenarios, the use of efavirenz was not significantly associated with the risk of depressive disorders. On the other hand, when only the diagnosis of depressive disorders made by psychiatrists were considered in the analysis, the use of efavirenz was significantly associated with a reduced aHR of depressive disorders (aHR = 0.76, 95% CI, 0.61–0.96, *p* = 0.018). Additionally, the three sensitivity analyses all indicated that both a past history of psychiatric disorders and a lower monthly income level were significant risk factors for depressive disorders among HIV-infected patients.

## 4. Discussion

In this 11-year population-based follow-up study in Taiwan, we found that the incidence rates of depressive disorders were not significantly associated with the use of efavirenz in a real-world setting. We found that HIV-infected patients with a past history of psychiatric disorders other than depressive disorders were more likely to develop depressive disorders during the follow-up. Similar findings were reported in a previous French study, which found the risk of developing depressive disorders among patients who presented a past history of depressive disorders to be five times higher than among those without the same disorders [18].

Our study results are generally consistent with findings from previous studies. In a study in Spain with a six-year follow-up, the authors noted an incidence rate of depression of 7.06 per 1000 PY in people living with HIV after ART was initiated [20]. The Spanish cohort found that the use of efavirenz was not associated with an increased risk of clinically significant depression. Instead, the study emphasized that the use of ART, no matter what kind of regimen, significantly decreased the incidence of depressive disorders [20]. The frequency of anti-depressants use was also not significantly different between patients who were and were not taking efavirenz in a nationwide 14-year follow-up in Denmark [11]. A study using the depression scale of the Patient Health Questionnaire (PHQ-9) to examine the development of depression associated with ART use also demonstrated baseline depression as an independent risk for 12-month depression, while an efavirenz-based regimen did not show such association [23].

The occurrence of depressive disorders among HIV-infected patients is complex, and the possible pathophysiology is unclear. Some researchers have suggested a bidirectional association between HIV infection and depression, and the mechanisms included biological, psychological, and social factors, which probably interact with each other [24]. Several biological models have been proposed; for instance, the prolonged activation of infected immune system cells resulted in the release of toxic viral proteins and an increased release of inflammatory cytokines [24,25]. Recent studies have also reported that T-cells could be activated under certain conditions via the sigma-1 signaling pathway, further enhancing the replication of HIV and increasing monocyte adhesion/transmigration into the central nervous system (CNS) [26,27]. However, other studies have proposed the hypothesis that the pro-inflammatory cytokines could increase the activity of tryptophan-2-3-dioxygenase, reducing serotoninergic neurotransmission in the brain and possibly leading to depressive symptoms, while efavirenz-treated mice expressed reduced TPO activity and higher serotonin against the occurrence of depression [28,29]. It is still necessary to investigate the effect of efavirenz on the CNS system. By understanding the pathophysiology of the development of depressive disorders among HIV-infected patients, targeted therapies could be applied as treatment alternatives, as in other neuropsychiatric disorders [30,31].

In the final scenario, our sensitivity analysis defined the occurrence of depressive disorders only when the psychiatrists had made the diagnoses. Aside from risk factors such as lower monthly income, living in Southern Taiwan, and a past history of psychiatric disorders other than depressive disorders, the use of efavirenz has shown a significantly reduced aHR for developing depressive disorders. The reason for this result is unclear. We speculated that the psychiatrists might not have made the diagnosis for depressive disorders upon learning that patients had been using efavirenz. Whether the observed protective effect from the use of efavirenz is real or not requires further studies.

In this study, we also found a geographic difference in the HR of depressive disorders among HIV-infected patients in Taiwan. Patients living in Southern Taiwan were at higher risks of developing depressive disorders, which is probably because HIV-infected patients in Southern Taiwan were more suppressed by social stigma related to concerns over HIV infection itself and sexual orientation [32]. Further studies are warranted to explore the underlying reasons for such phenomena. Besides, patients with higher monthly income were less likely to have depressive disorders, which was consistent with other studies regarding depression among HIV-infected patients [10].

Some strengths are noted in this study. This was a population-based study that included nearly all HIV-infected patients in Taiwan. A large number of administrative claim data spanning an 11-year period was used to examine the associations between the use of efavirenz and the occurrence of depressive disorders. The administrative claims database allowed for the assessment of associations, with little likelihood of recall bias. Additionally, the information on the duration of efavirenz use could be obtained from the database to set up a more accurate exposure group. Moreover, with the long observational period, the occurrence of depressive disorders could be detected to the greatest extent, fully addressing the long-term effects of efavirenz. Treatment guidelines around the world nowadays suggest fewer non-nucleoside reverse transcriptase inhibitor-based regimens than previous guidelines did [33,34]. Studies addressing issues caused by newer drugs, regardless of whether they are 2-drug regimens or 3-drug regimens, might still be warranted in the future.

Our study had several limitations. Firstly, the diagnosis of depressive disorders was based solely on inpatient and outpatient claims; as such, disease coding errors are inevitable. However, in our study, around three-fourths of the diagnoses of depressive disorders had been made by psychiatrists, which may help provide reassurance for the accuracy of diagnosis. Second, the presence of prescriptions for certain medications in the claims did not guarantee that those medications were actually taken by patients in need of them. Furthermore, other ARTs, such as nucleoside reverse transcriptase inhibitors as backbone drugs, might still have certain uncovered adverse effects as potential confounders to this study. Such potential drug exposure misclassification could happen to both the exposed and non-exposed groups. Such potential drug exposure misclassification is likely to be non-different, which may have reduced the association between efavirenz and depressive disorders. Third, some important clinical or laboratory parameters are not available from the NHI medical claims, such as CD4 lymphocyte counts and viral load. Due to the lack of these variables, we were unable to determine the disease severity in the HIV-infected patients, which further limited our interpretation of the study findings. Additionally, information on lifestyle characteristics was also unavailable from the NHI data, which resulted in the potential for confounding in our study. Nonetheless, we did include some major comorbidities, especially opportunistic infections, as a representative of disease severity for adjustment as a confounder. Finally, the tendency to prescribe efavirenz or not is diverse among different specialists. It is almost impossible to have a standard for the prescription of ART. Some physicians would defer the use of efavirenz for patients with a past history of depressive disorders or psychiatric diseases. In an attempt to assess the potential for confounding by indication, we used age at HIV diagnosis, gender, and past history of psychiatric disorders (other than depressive disorders) to calculate the propensity score for the physicians’ treatment assignment, but there were no obvious differences in the distribution of the propensity score between the two comparison groups.

## 5. Conclusions

In conclusion, this population-based study found that efavirenz use was not significantly associated with an increased risk of depressive disorders among HIV-infected patients. However, patients with a past history of psychiatric disorders (other than depressive disorders) and those with lower income levels were associated with a higher risk of depressive disorders. Detailed history-taking before the prescription of ARTs is essential for clinicians, and vigilance with regard to these diseases should be raised for certain higher risk patients.

## Figures and Tables

**Table 1:** Demographic characteristics of HIV-infected patients, with or without efavirenz therapy, 2000–2009.

Variables	Efavirenz-Treated Group (*n* = 4047)	Non-Efavirenz Treated Group (*n* = 3239)	*p* Value
Gender			0.282
Male	3777 (93.3)	3043 (93.9)	
Female	270 (6.7)	196 (6.1)	
Age of HIV diagnosis, years	35.0 (20–81, 10.4)	35.1 (20–81, 10.6)	0.658
20–29	1453 (35.9)	1209 (37.3)	0.554
30–39	1561 (38.6)	1191 (36.8)	
40–49	654 (16.2)	521 (16.1)	
50–59	257 (6.3)	219 (6.7)	
≥60	122 (3.0)	99 (3.1)	
Residence area			<0.001
North	2109 (52.1)	2037 (62.9)	
Central	667 (16.5)	455 (14.0)	
South	1121 (27.7)	628 (19.4)	
East and outer islands	150 (3.7)	119 (3.7)	
Insurance premium based monthly income, NTD			0.005
≤17,280	1940 (47.9)	1488 (45.9)	
17,281–26,400	1058 (26.2)	790 (24.4)	
26,401–43,900	649 (16.0)	591 (18.3)	
≥43,901	400 (9.9)	370 (11.4)	
Comorbidities			
Opportunistic infections	2268 (56.0)	1675 (51.7)	<0.001
Hypertension	147 (3.6)	129 (4.0)	0.436
Diabetes mellitus	93 (2.3)	84 (2.6)	0.416
Cardiovascular diseases	26 (0.6)	32 (0.9)	0.099
Psychiatric disorders (other than depressive disorders)	239 (5.9)	208 (6.4)	0.362
Observation time, mean (range, SD), years	6.1 (0–11, 3.9)	4.7 (0–11, 3.16)	<0.001
Depressive disorders	304 (7.5)	191 (5.9)	<0.001

NTD = New Taiwan Dollar; SD = Standard deviation.

**Table 2:** Cox proportional hazard model for hazard ratio of depressive disorder onset among 7286 HIV-infected patients.

	No. of Depressive Disorders/Total No.	Crude		Adjusted	
		RR (95% CI)	*p* Value	HR (95% CI)	*p* Value
Efavirenz use					
Yes	304/4047	1.25 (1.05–1.50)	0.011	0.92 (0.76–1.10)	0.353
No	191/3239	1.00		1.00	
Gender					
Male	459/6820	1.00		1.00	
Female	36/466	1.14 (0.82–1.58)	0.441	0.81 (0.57–1.14)	0.222
Age of HIV diagnosis, years					
20–29	189/2662	1.00		1.00	
30–39	190/2752	0.97 (0.80–1.18)	0.792	0.88 (0.72–1.08)	0.225
40–49	65/1175	0.79 (0.60–1.04)	0.093	0.79 (0.60–1.06)	0.113
50–59	37/476	1.09 (0.77–1.53)	0.627	0.85 (0.59–1.23)	0.738
≥60	14/221	0.90 (0.53–1.52)	0.691	0.58 (0.33–1.02)	0.056
Insurance premium-based monthly income, NTD					
≤17,280	304/3428	2.04 (1.43–2.92)	<0.001	2.59 (1.79–3.76)	<0.001
17,281–26,400	114/1848	1.46 (0.99–2.14)	0.054	1.55 (1.04–2.31)	0.030
26,401–43,900	45/1240	0.88 (0.56–1.37)	0.565	0.94 (0.59–1.48)	0.774
≥43,901	32/770	1.00		1.00	
Residence area					
Northern	259/4146	1.00		1.00	
Central	81/1122	1.15 (0.90–1.46)	0.271	1.24 (0.96–1.59)	0.101
Southern	139/1770	1.24 (1.02–1.51)	0.035	1.49 (1.21–1.84)	<0.001
Eastern and outer islands	16/248	1.03 (0.63–1.68)	0.903	0.99 (0.60–1.64)	0.966
Comorbidities					
Opportunistic infections	245/3943	0.85 (0.71–0.99)	0.046	0.80 (0.67–0.95)	0.011
Hypertension	18/276	0.96 (0.61–1.52)	0.864	1.21 (0.72–2.02)	0.476
Diabetes mellitus	10/177	0.84 (0.46–1.54)	0.568	1.03 (0.54–1.97)	0.926
Cardiovascular diseases	3/58	0.77 (0.26–2.33)	0.646	1.29 (0.40–4.14)	0.669
Psychiatric disorders (other than depressive disorders)	67/447	2.21 (1.74–2.82)	<0.001	4.59 (3.51–6.01)	<0.001

RR = relative risk; HR = hazard ratio; CI = confidence interval; NTD = New Taiwan Dollar.

**Table 3:** The sensitivity analyses according to three scenarios.

	Scenario 1 ^a^			Scenario 2 ^b^			Scenario 3 ^c^		
	aHR	95% CI	*p* Value	aHR	95% CI	*p* Value	aHR	95% CI	*p* Value
Efavirenz use	0.83	0.68–1.01	0.068	0.97	0.81–1.17	0.744	0.76	0.61–0.96	0.018
Monthly insurance premium, NTD									
≤17,280	2.93	1.93–4.44	<0.001	2.93	1.93–4.44	<0.001	2.53	1.62–3.97	<0.001
17,281–26,400	1.71	1.09–2.67	0.020	1.72	1.10–2.68	0.018	1.63	1.01–2.64	0.045
26,401–43,900	0.96	0.57–1.61	0.867	0.96	0.57–1.61	0.870	0.94	0.54–1.63	0.812
≥43,901	1.00			1.00			1.00		
Residence area									
Northern	1.00			1.00			1.00		
Central	1.14	0.86–1.52	0.366	1.14	0.86–1.52	0.365	1.14	0.82–1.58	0.451
Southern	1.36	1.07–1.73	0.014	1.36	1.06–1.73	0.014	1.54	1.17–2.02	0.002
Eastern and outer islands	0.92	0.51–1.64	0.765	0.94	0.53–1.69	0.844	1.15	0.62–2.13	0.650
Opportunistic infections	0.88	0.72–1.07	0.204	0.88	0.72–1.07	0.187	0.89	0.71–1.11	0.292
Psychiatric disorders other than depression	4.07	2.96–6.00	<0.001	4.08	2.97–5.61	<0.001	4.89	4.47–6.89	<0.001

^a^ Patients who developed depressive disorders after the medication had been stopped for more than 1 month and whose efavirenz was replaced by other antiretroviral agents were excluded from analysis. ^b^ Patients who had not been prescribed efavirenz for more than six months were further excluded. ^c^ Patients whose depressive disorders had been diagnosed by psychiatrists were included in the analysis.

## Data Availability

Restrictions apply to the availability of these data. Data was obtained from the Bureau of National Health Insurance with the permission of the National Health Research Institutes.
